# Migraine triggers in Asian countries: a narrative review

**DOI:** 10.3389/fneur.2023.1169795

**Published:** 2023-05-03

**Authors:** Chisato Iba, Seiya Ohtani, Mi Ji Lee, Sunjun Huh, Narumi Watanabe, Jin Nakahara, Kuan-Po Peng, Tsubasa Takizawa

**Affiliations:** ^1^Department of Neurology, School of Medicine, Keio University, Tokyo, Japan; ^2^Department of Neurology, Seoul National University Hospital, Seoul National University College of Medicine, Seoul, Republic of Korea; ^3^Department of Systems Neuroscience, University Medical Center Hamburg-Eppendorf, Hamburg, Germany

**Keywords:** Asia, migraine, triggers, sleep, stress

## Abstract

**Background:**

Migraine is one of the most common neurological disorders worldwide. Clinical characteristics of migraine may be somewhat different across ethnic groups. Although factors such as stress, lack of sleep, and fasting are known as migraine triggers, the discussion about geographical differences of migraine triggers in Asia is lacking.

**Methods:**

In this study, we performed a narrative review on migraine triggers in Asia. We searched PubMed for relevant papers published between January 2000 and February 2022.

**Results:**

Forty-two papers from 13 Asian countries were included. Stress and sleep are the most frequently reported migraine triggers in Asia. There were some differences in migraine triggers in Asian countries: fatigue and weather common in Eastern Asia and fasting common in Western Asia.

**Conclusion:**

Majority of the common triggers reported by patients with migraine in Asia were stress and sleep, similar to those reported globally, thus showing they are universally important. Some triggers linked to internal homeostasis are influenced by culture (e.g., alcohol, food/eating habit), and triggers related to environmental homeostasis, such as weather, are highly heterogenous between regions.

## 1. Introduction

Migraine is one of the most common neurological disorders worldwide, affecting over 1 billion people (1). The prevalence of migraine globally is about 10% (6% for males and 14% for females). However, the prevalence varies across regions: it is higher in Europe (15%) and North America (13%), while it is lower in Asia (9%) and Africa (5%) (2, 3). However, the prevalence of migraine may be higher in Asia when probable migraine is included, according to a Korean study in which the total prevalence of definite migraine and probable migraine was 17.5% (4). This may suggest different clinical characteristics of migraine among Asians, considering that the current definition of migraine is primarily based on European and American studies. For example, Asians are generally known to have a lower prevalence of aura (5, 6) and a higher prevalence of osmophobia (7, 8). In addition, given the diversities in culture, religion, and geography within Asia, it is reasonable to compare different population groups within Asia.

The common migraine triggers reported by patients include stress, lack of sleep, fasting, sensory stimuli (such as auditory stimuli), and hormonal changes (9–11). It is important to focus on triggers since knowing and avoiding them can sometimes prevent a migraine attack. Regional differences in migraine triggers may possibly arise from different exposures that could be migraine triggers. We queried whether migraine triggers among Asians differ from those reported in other parts of the world. In addition, we aimed to investigate differences in migraine triggers according to diversities in geography, culture, and religion in Asia. Although several papers have reported triggering factors for migraine in each country, research on regional differences is lacking. Despite Asia having a population of more than 4.5 billion, no narrative review has been performed on migraine triggers in this region. In this study, we focused on triggers that were queried in each study, reflecting what is thought as a trigger, and the actual proportion of patients reporting a particular trigger.

## 2. Methods

**Figure 1:**
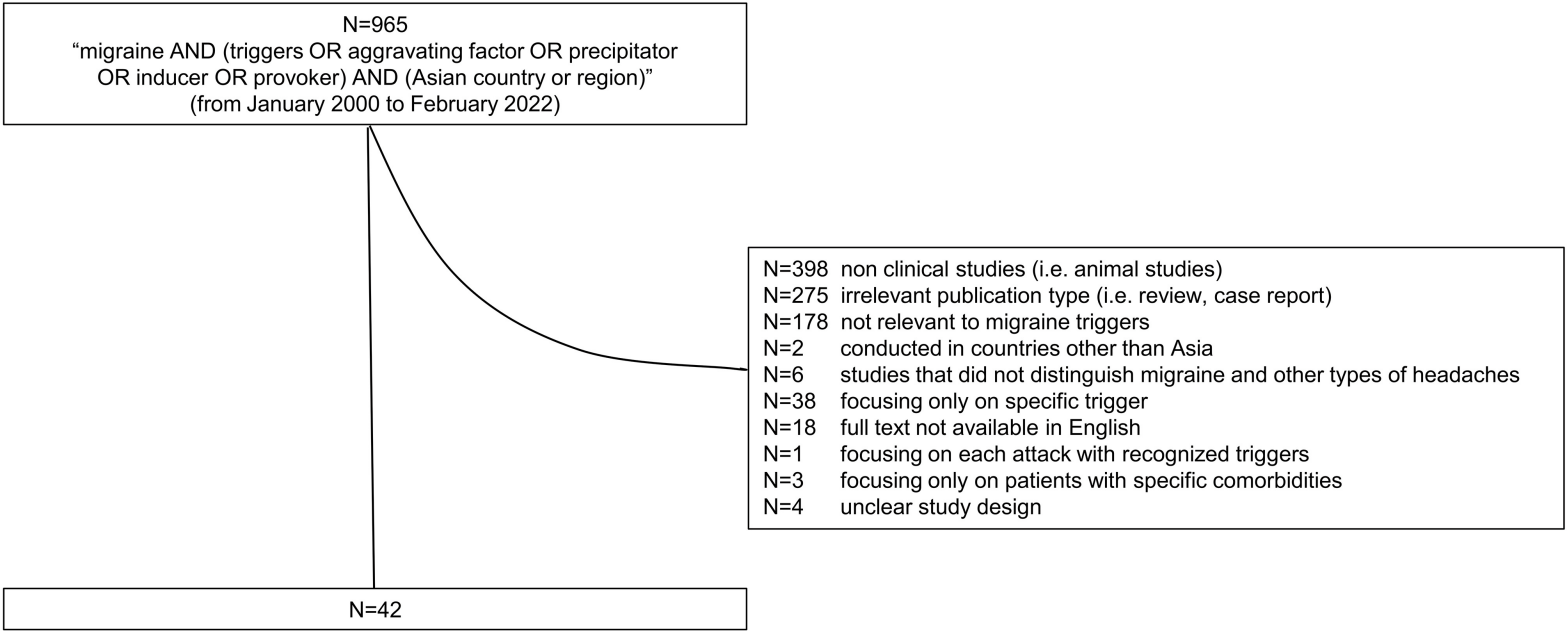
The flowchart of the narrative review. The inclusion and exclusion criteria of the study are shown.

**Figure 2:**
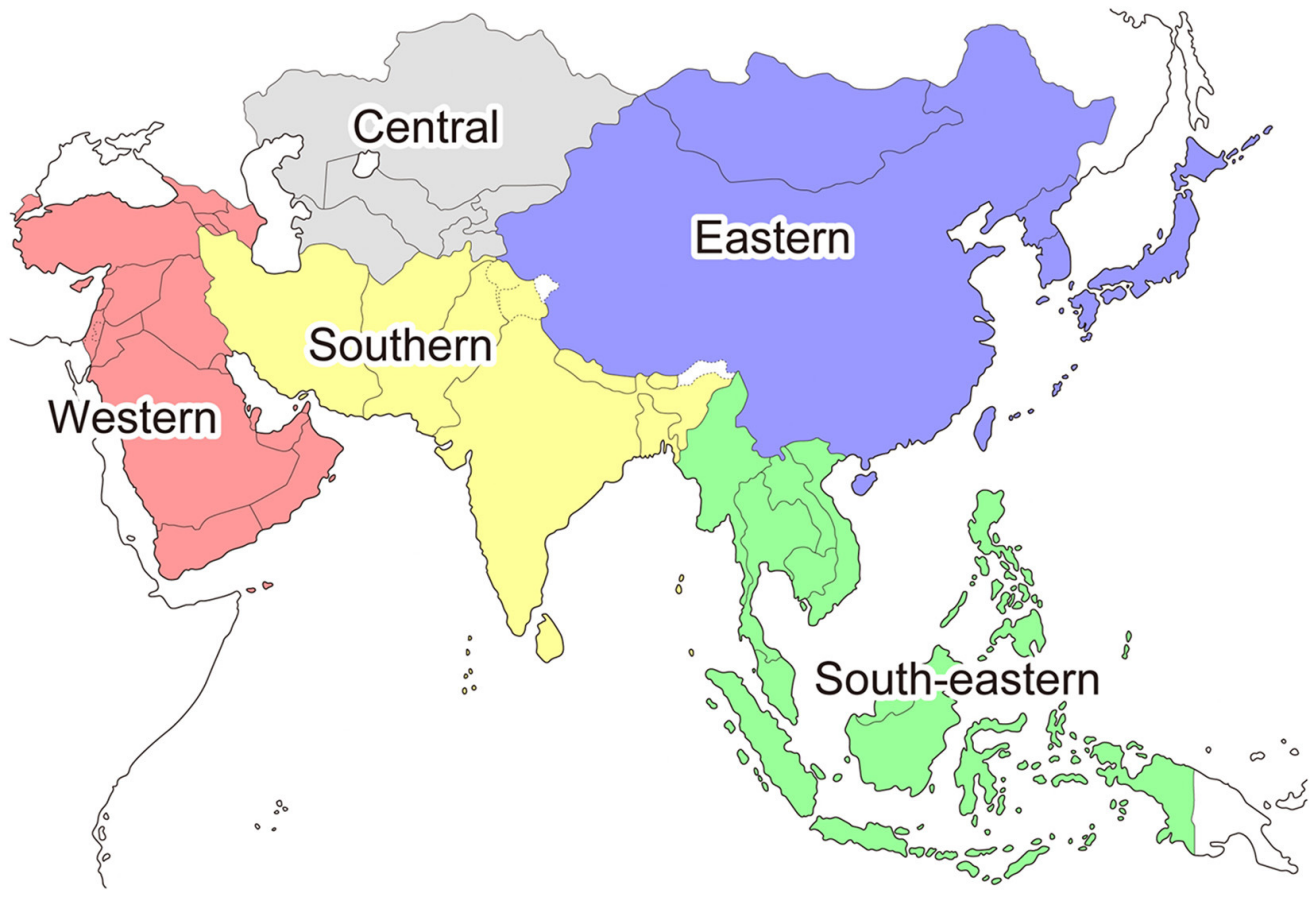
The categorization of Asian countries based on the United Nations Statistics Division.

We searched PubMed database according to the flowchart shown in Figure 1. As of February 2022, the following keywords were entered into the field search box; migraine AND (triggers OR aggravating factor OR precipitator OR inducer OR provoker) AND (the name of Asian country or region) AND “2000/01/01” [edat]: “2022/02/28” [edat]. Relevant studies published after January 1, 2000, and those conducted in Asian countries were included in this study. All these procedures were conducted by one author (C.I.) and double-checked by other authors (S.O., S.H.). Our exclusion criteria were non clinical studies, irrelevant publication type, those not relevant to migraine triggers, conducted in countries other than Asia, studies that did not distinguish migraine and other types of headaches, focusing only on specific trigger, full text not available in English, focusing on each attack with recognized triggers, focusing only on patients with specific comorbidities, and unclear study design (Figure 1). Migraine triggers were categorized into 15 groups based on a meta-analysis by Pellegrino et al. (9). Asian countries were divided into five different regions: Central Asia, Eastern Asia, South-eastern Asia, Southern Asia, and Western Asia. This categorization was adapted from the geographic regions by the United Nations Statistics Division (12) (Figure 2).

The percentages for each trigger were calculated as follows: (a) the number of respondents that recognized each factor as a trigger was divided by the number of all patients in each study, and (b) the number of patients who answered that triggers were related to menstruation were divided by the total number of female patients accordingly.

## 3. Results

Forty-two studies (13–54) from 13 countries were included. Twelve studies were from India, 10 studies from Turkey, four from Saudi Arabia, three from Bangladesh, two each from China, Japan, Pakistan, and Thailand, and one each from Iraq, Kuwait, Lebanon, Philippines, and UAE. We were not able to find studies from countries in Central Asia.

**Figure 3:**
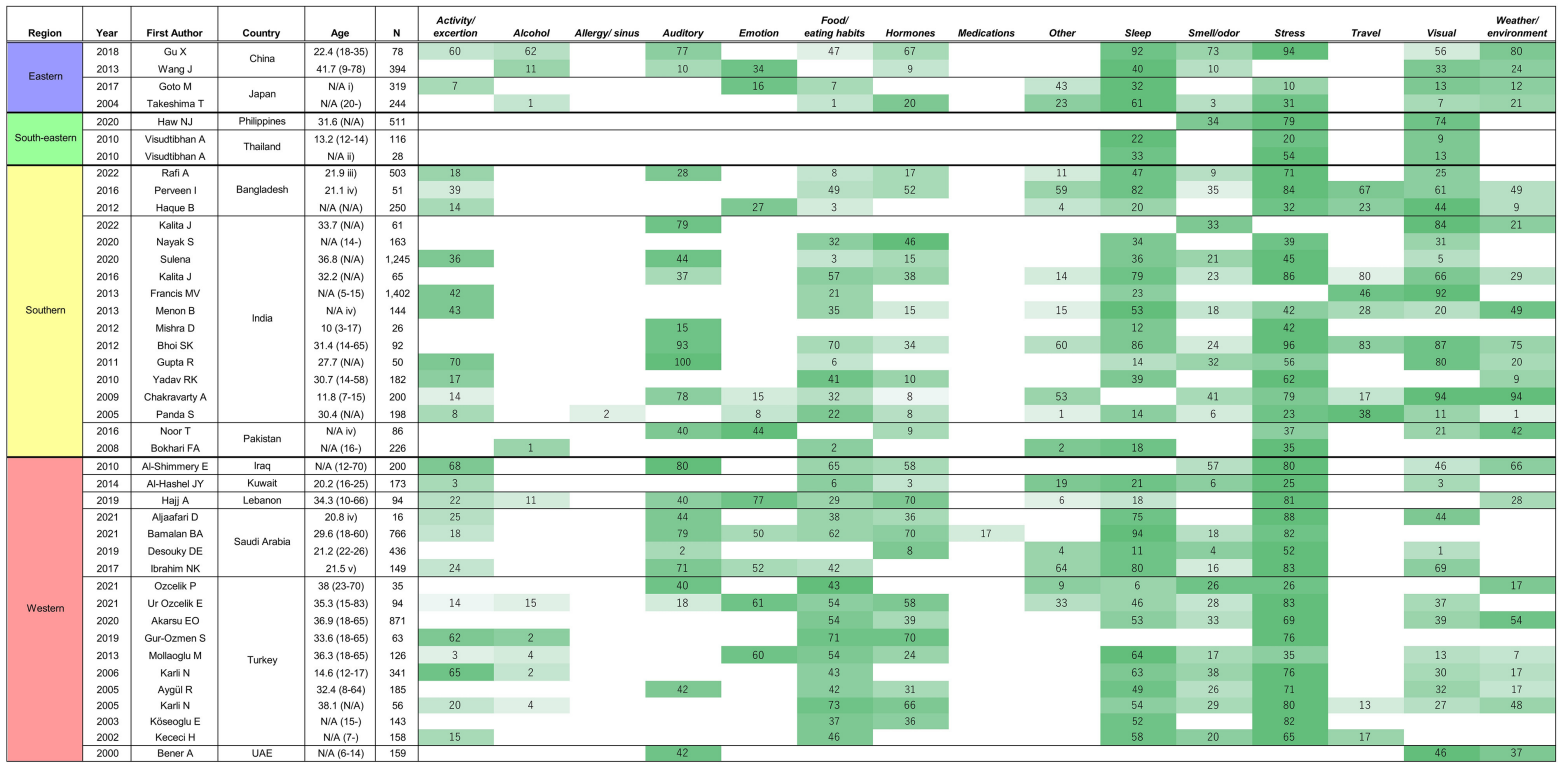
The prevalence of migraine triggers in Asian countries. Countries are listed in order of regions in Asia: Eastern Asia, South-eastern Asia, Southern Asia, and Western Asia. When multiple items belonged to a group, the highest proportion was applied to the figure. The assigned colors are determined according to the ranking in each study (i.e., darker colors represent factors recognized by more respondents). Age was not available in some studies. The provided information about the background of the studied group is shown below [age; mean (range)]. N/A, not available, (i) elementary/junior high school students, (ii) junior high school students, (iii) university students, (iv) medical students, (v) medical students and interns.

Table 1 shows 15 categories and relevant triggers in each group. Figure 3 shows the percentage of patients that recognized each categorized factor as a migraine trigger and its ranking in each study. When multiple items belonged to a group, the highest proportion was applied to the figure. Most of the factors categorized into “Others” were related to hygiene (i.e., bathing, hair wash) and reading. Triggers related to reading activities were common in Southern and Western Asia, such as Bangladesh, India, Kuwait, and Saudi Arabia (21, 33, 38, 42). Eleven studies reported a percentage of 7.2% for migraineurs who did not recognize any triggers (14, 17, 19, 28, 29, 32, 33, 36, 37, 44, 53). The three most frequent triggers in each study are shown in the Supplementary Table 1.

**Table 1:** Fifteen categories of migraine triggers and included terms in each group.

Activity/exertion	Activity, daily physical activity, exertion, exercise, head and neck movement, head bending, head movements, physical activity, physical exercise, sports, strenuous physical exercises like dancing and cycling
Alcohol	Alcohol, wine
Allergy/sinus	Dust, pollution
Auditory	Environmental noise, environmental noise (e.g., loudspeaker, crowd etc.), listening to talks, audit. overexposure, high noise, noise, loud noise, sounds
Emotion	Aggressiveness, anxiety, behavioral (shouting), depression, negative affect, negative feeling
Food/eating habits	Certain diet, coffee, consumables and eatables, fasting, fasting < 12 h, fasting or missing food, fasting or starving, food, foods, hunger, ice cream, missed meal, missing a meal, missing meal at the right time, missing meals, relation to Ramadan & fasting, specific food or drink (e.g., coffee, tea, chocolates etc.)
Hormones	Catamenial, hormonal factors, hormonal factors such as menstruation, menses, menses (among females), menstrual cycle, menstrual period, menstrual periods, menstruation, menstruation in female
Medications	Drug consumption
Other	Bath, bathing, dry hair, extended reading hours, hair wash, katakori, lipstick, much reading, others, others (perfumes, head trauma, GI upset), reading, reading or general thinking, school book reading, thinking/concentration, weekend
Sleep	Changes in sleep, excess sleep, fatigue, inadequate sleep, irregular sleep, lack of sleep, sleep deprivation, sleep disturbance, sleep disturbances, sleep disturbances, sleep-in, sleeplessness,
Smell/odor	Irritant smell, odor, odors, perfume, petrol, smell, smell (perfume, gasoline, food and cleaness product), smells, smoke, smoke (auto emission), smoking, smoking or shisha consumption, smoking or cigarette smoke, special taste or aroma, strong odors, strong smell
Stress	Academic stress, conflict, emotional stress, exam stress, mental stress, psychological upset, school work, stress, stress: related to school activities, stress and anxiety, stress at study work, stress or anxiety, stress (anxiety), stress/heavy workload, stress/tension, study related stress, tension
Travel	Altitude, car/bus journeys (other than school), frequent prolong traveling, journey, travel, traveling, traveling by bus
Visual	Alternative light, bright light, clarity, computer related, electronic device use (e.g., laptop, mobile phones), exposure to sun, exposure to sunlight, flickering lights, light, looking at computer screen for too long, playing at the computer, prolonged computer use, sun. exposure, sun/clarity, sunlight, TV games, use of computers, using PC, visual stimuli, shade, watching TV, work on computer
Weather/environment	Change in weather, changes in weather, change of environment, cloudy weather, cold, cold air, excessive environment with bright light, heat, hot and humid climate, hot climate, hot exposure, hot weather, humidity, sudden change in temperature, summer, sun/ heat, weather change, weather/climate, wind

Migraine triggers were categorized in 15 groups based on a review by Pellegrino et al. (9). The triggers included in each category are listed.

Among the 15 categories, *Stress* was the most frequently queried factor in the included studies (90%), followed by *Sleep* and *Food/eating habits* (83 and 79%, respectively). In contrast, the following categories were the least investigated: *Allergy/sinus* (2%), *Medications* (2%), *Travel* (24%), and *Alcohol* (24%). In addition, 66% of the investigating studies reported that *Stress* was the leading trigger, and 74% reported that *Sleep* was among the third leading triggers. Overall, migraine triggers that were frequently queried had a higher prevalence (i.e., *Stress, Sleep*) compared to the ones that were infrequently asked about (i.e., *Travel* and *Alcohol*), although there were some exceptions, such as *Hormones*, which showed lower prevalence as frequent triggers despite higher awareness.

We noted several differences in migraine triggers depending on the regions in Asia. In Eastern Asia, *Sleep* was observed to be more frequent than *Stress*, although *Stress* is more prevalent in Asia as a whole. Furthermore, fatigue is listed frequently in this region as a major triggering factor (15, 16). Another characteristic in this region is the higher awareness of *Weather/environment* as a trigger. *Weather/environment* was investigated by all studies from Eastern Asia, compared to 57% of studies in Asia (13–16). In South-eastern Asia, only three papers focused on migraine trigger. In total, only four categories of triggers were queried, but all papers from South-eastern Asia investigated *Stress* and *Visual* triggers (17–19). Southern Asia has high variations in the leading factors compared to other regions in Asia. For example, *Alcohol* was queried in only one (6%) study by Bokhari et al. compared to 24% of studies in entire Asia. The study by Panda et al. is the only paper investigating *Allergy* in Asia. In India, *Auditory* is ranked relatively higher compared to other countries. Eight out of 10 papers investigating *Travel* are from Southern Asia (20–36). In Western Asia, *Food/eating habits* were the common triggering factors compared to other areas in Asia (37–54).

## 4. Discussion

This study focused on migraine triggers in Asian countries. It is evident that there are high prevalence and awareness in *Stress* and *Sleep* triggers, similar to the trend reported globally in the 2018 meta-analysis by Pellegrino et al. (9). Furthermore, the current study has demonstrated that regional differences exist in Asia, partially reflecting the geographical and cultural diversity in a continent that covers 44.58 million km^2^ and with a population of 4.5 billion.

Regional differences in migraine triggers may be accounted for by the study design and cultural differences. In Eastern Asia, fatigue is often reported as a top three migraine triggers. Furthermore, *Weather/environment* was a highly recognized trigger in Eastern Asian countries (13–16). This may reflect distinct seasonal changes in barometric pressure, temperature, and humidity in these regions compared with the other Asian regions (55). The latest report using a Japanese smartphone application revealed that low barometric pressure, barometric pressure changes, higher humidity, and rainfall, all of which are characteristic to the climate in Japan, are associated with an increased number of headache occurrences (56).

In Western Asia, where the majority of the population is Muslim, *Food/eating habits*, especially hunger, were highly reported as migraine triggers compared with other countries in Asia. Some papers investigate about specific food/beverage as migraine triggers such as coffee, cheese, chocolate (39, 45, 47, 48), which are well-known migraine triggers, but mostly nothing particular for food/beverage in the region. Rather, most of papers from Western Asia focused on food as a whole and/or hunger. This is thought to be associated with Ramadan, the month of fasting, one of the five fundamental practices in Islam (37–54). A recent study reported a significant increase in migraine frequency during the month of Ramadan compared with the previous month (57). In addition, nearly 90% of patients with migraine in the Kuwait cohort study reported changes in sleep and food habits during the month of Ramadan (57). Ragab et al. have also reported that migraine frequencies among Egyptian patients were significantly increased during the first 10 days of Ramadan but subsequently normalized during the rest 20 days of Ramadan (58). Although there is insufficient scientific evidence on the mechanisms by which fasting or post-prandial after fasting triggers migraine, it has been suggested that a change in the usual dietary routine itself could be the migraine trigger and aggravating factor (57, 58). Recent studies have shown that alteration in hypothalamic-pituitary-adrenal axis is involved in pathologies of migraine attacks (59). Therefore, the relatively high prevalence of triggers related to *Food/eating habits* in Western Asia may be related to Ramadan.

Sensitivity to light and/or sound is one of the premonitory or accompanied symptoms of migraine (60). Factors related to *Visual*, such as bright light, and *Auditory*, such as noise, are recognized as migraine triggers (79% and 50% of studies queried for the presence of the triggers, respectively). Reported migraine triggers in such categories may possibly include not only triggers of headache but also prodromal symptoms, which are difficult to distinguish from the real triggers. Schulte et al. reported that factors such as visual and auditory stimuli were not actual migraine triggers but misunderstood symptoms of migraine attacks (61).

In European/North American countries, about 30–40% of patients with migraine reported that alcohol or wine was the triggering factor (62). However, this proportion tended to be lower in Asian countries (Figure 3), implying that alcohol is less investigated or less likely to be a trigger in this region. In European/North American countries, alcohol consumption per capita is mostly higher than that in Asian countries (63). Religious customs of Muslims and Hindus may be related to the lower alcohol consumption in the Asian population, which results in lower prevalence of alcohol as a recognized migraine trigger in Asia.

Differences in race are the factors which should not be ignored on discussion about differences in migraine triggers. Asia is muti-ethnic region. For example, Southern Asia consists Persian and Hindus where Western Asia is composed of Caucasians and Arabs. Even in the same geographical area, there are differences in migraine triggers. For example, *Food/eating habits* are common in Turkey, while *Auditory* is common in Saudi Arabia. Turkey consists of Turkish, and Saudi Arabia consists of Arab, and the genetic differences may be related to the sensitivity to each migraine trigger in addition to geographical differences.

It is evident that even in the same country, results of migraine triggers varied depending on specific studies (Figure 3). The migraine triggers are strongly biased by the study design: you only get what you ask for. Certain studies did not cover the commonly reported triggers in other studies or provided only a few options to select. This suggests that in addition to the original/innate geographical and racial differences in migraine triggers, bias due to the research design, such as differences in languages and the approach of asking about migraine triggers in questionnaires, should not be ignored.

One way to reduce such bias and clarify the geographical or racial characteristics in migraine triggers may be to conduct a multicenter study or a survey targeting population groups. Ur Özçelik et al. (45) conducted a multicenter study in Turkey, Brazil, Guatemala, and Lithuania and reported notable differences in migraine triggers among the countries (i.e., chewing in Lithuania, physical stress in Brazil, and pleasant tastes in Turkey showed a higher migraine-provoking rate than in other countries). Carod-Atral et al. conducted a cross-cultural study in Spain and Brazil and reported that Brazilians, compared to Spanish, were more likely to consider a condition as a trigger in nearly all categories, such as stress and sleep disturbances. In this study, the most recognized trigger in Brazil was sleep disturbances, followed by menstruation and smells/odor. In contrast, stress, followed by menstruation and sleep disturbances, were the recognized triggers among the Spanish (64). Tai et al. (65) conducted a survey in Malaysia, with respondents comprising Malays, Chinese, and Indians, and identified that Malays had more dietary triggers than Indians. This may also be related to Ramadan because most Malays are Muslims. Goadsby et al. surveyed characteristics and triggers of migraine in five countries using a smartphone application. In Japan, the only Asian country included in this study, neck pain and weather seemed more frequent triggers compared to the other countries (66). These studies suggest that conducting the same survey in multiple countries or for multiple ethnic groups will make it possible to focus on racial and environmental differences without considering the differences in research designs.

This study has several limitations. First, we gathered studies focusing on migraine triggers irrespective of the different study designs. In addition, we did not include papers focusing solely on specific triggers (i.e., focusing solely on alcohol consumption). Moreover, studies included in this paper are not enough to come to the conclusion about the effect of genetic differences in the similar geographical area. Furthermore, we did not compare Asian studies with individual studies from other regions across the world.

In conclusion, the most common migraine triggers in Asia are stress and sleep, similar to most studies globally. Stress and sleep are universally important migraine triggers because they are related to internal homeostasis, while others such as alcohol and food/eating habits, are influenced by culture and religion. Triggers related to environmental homeostasis, such as weather, are highly heterogenous between regions. Fatigue and weather are prevalent in Eastern Asia, while fasting is dominant in Western Asia. In future, large-scale international joint research will be necessary to understand more about regional and racial differences in migraine triggers. Cross-national cooperation may not only facilitate direct comparison between different regions but may also reduce bias secondary to incongruent study design.

## Author contributions

CI: investigation, visualization, and writing. SO, SH, and NW: investigation and editing. ML, JN, and K-PP: supervision and editing. TT: conceptualization, supervision, writing, editing, and funding acquisition. All authors contributed to the article and approved the submitted version.

## Supplementary material

Supplementary Table 1 Three most frequent triggers in each study. Age was not available in some studies. The provided information about the background of the studied group is shown below [age; mean (range)]. N/A, not available, (i) elementary/junior high school students, (ii) junior high school students, (iii) university students, (iv) medical students, (v) medical students and interns.

## References

[1] GBD. Headache collaborators. Global, regional, and national burden of migraine and tension-type headache, 1990–2016: a systematic analysis for the Global Burden of Disease Study 2016. Lancet Neurol. (2018) 17:954–76. 10.1016/S1474-4422(18)30322-3 30353868 PMC6191530

[2] Ashina M Katsarava Z Do TP Buse DC Pozo-Rosich P Özge A. Migraine: epidemiology and systems of care. Lancet. (2021) 397:1485–95. 10.1016/S0140-6736(20)32160-7 33773613

[3] Robbins MS Lipton RB. The epidemiology of primary headache disorders. Semin Neurol. (2010) 30:107–19. 10.1055/s-0030-1249220 20352581

[4] Kim BK Chung YK Kim JM Lee KS Chu MK. Prevalence, clinical characteristics and disability of migraine and probable migraine: a nationwide population-based survey in Korea. Cephalalgia. (2013) 33:1106–16. 10.1177/0333102413484990 23615490

[5] Alders EE Hentzen A Tan CT. A community-based prevalence study on headache in Malaysia. Headache. (1996) 36:379–84. 10.1046/j.1526-4610.1996.3606379.x 8707557

[6] Wang SJ Fuh JL Young YH Lu SR Shia BC. Prevalence of migraine in Taipei, Taiwan: a population-based survey. Cephalalgia. (2000) 20:566–72. 10.1046/j.1468-2982.2000.00085.x 11075840

[7] Wang YF Fuh JL Chen SP Wu JC Wang SJ. Clinical correlates and diagnostic utility of osmophobia in migraine. Cephalalgia. (2012) 32:1180–8. 10.1177/0333102412461401 23038716

[8] Saisu A Tatsumoto M Hoshiyama E Aiba S Hirata K. Evaluation of olfaction in patients with migraine using an odour stick identification test. Cephalalgia. (2011) 31:1023–8. 10.1177/0333102411410612 21628440

[9] Pellegrino ABW Davis-Martin RE Houle TT Turner DP Smitherman TA. Perceived triggers of primary headache disorders: a meta-analysis. Cephalalgia. (2018) 38:1188–98. 10.1177/0333102417727535 28825314 PMC8544028

[10] Peroutka SJ. What turns on a migraine? A systematic review of migraine precipitating factors. Curr Pain Headache Rep. (2014) 18:454. 10.1007/s11916-014-0454-z 25160711

[11] Kelman L. The triggers or precipitants of the acute migraine attack. Cephalalgia. (2007) 27:394–402. 10.1111/j.1468-2982.2007.01303.x 17403039

[12] United Nations Statistics Division. Methodology. (2022). Available online at: https://unstats.un.org/unsd/methodology/m49/?msclkid=9c64e3cbb24211ec8f53a8dc5f9c6393 (accessed February 18, 2022).

[13] Gu X Xie Y. Migraine attacks among medical students in Soochow University, Southeast China: a cross-sectional study. J Pain Res. (2018) 11:771–81. 10.2147/JPR.S156227 29695929 PMC5905467

[14] Wang J Huang Q Li N Tan G Chen L Zhou J. Triggers of migraine and tension-type headache in China: a clinic-based survey. Eur J Neurol. (2013) 20:689–96. 10.1111/ene.12039 23356519

[15] Goto M Yokoyama K Nozaki Y Itoh K Kawamata R Matsumoto S. Characteristics of headaches in Japanese elementary and junior high school students: a school-based questionnaire survey. Brain Dev. (2017) 39:791–8. 10.1016/j.braindev.2017.05.010 28578816

[16] Takeshima T Ishizaki K Fukuhara Y Ijiri T Kusumi M Wakutani Y. Population-based door-to-door survey of migraine in Japan: the Daisen study. Headache. (2004) 44:8–19. 10.1111/j.1526-4610.2004.04004.x 14979878

[17] Haw NJ Cabaluna IT Kaw GE Cortez JF Chua MP Guce K. A cross-sectional study on the burden and impact of migraine on work productivity and quality of life in selected workplaces in the Philippines. J Headache Pain. (2020) 21:125. 10.1186/s10194-020-01191-6 33109071 PMC7590802

[18] Visudtibhan A Boonsopa C Thampratankul L Nuntnarumit P Okaschareon C Khongkhatithum C. Headache in junior high school students: types & characteristics in Thai children. J Med Assoc Thai. (2010) 93:550–7. 20524440

[19] Visudtibhan A Thampratankul L Khongkhatithum C Okascharoen C Siripornpanich V Chiemchanya S. Migraine in junior high-school students: a prospective 3-academic-year cohort study. Brain Dev. (2010) 32:855–62. 10.1016/j.braindev.2009.12.004 20060252

[20] Rafi A Islam S Hasan MT Hossain G. Prevalence and impact of migraine among university students in Bangladesh: findings from a cross-sectional survey. BMC Neurol. (2022) 22:68. 10.1186/s12883-022-02594-5 35219314 PMC8881749

[21] Perveen I Parvin R Saha M Bari MS Huda MN Ghosh MK. Prevalence of irritable bowel syndrome (IBS), migraine and co-existing IBS-migraine in medical students. J Clin Diagn Res. (2016) 10:OC09–13. 10.7860/JCDR/2016/20900.8832 28050419 PMC5198372

[22] Haque B Rahman KM Hoque A Hasan AT Chowdhury RN Khan SU. Precipitating and relieving factors of migraine versus tension type headache. BMC Neurol. (2012) 12:82. 10.1186/1471-2377-12-82 22920541 PMC3503560

[23] Kalita J Misra UK Bansal R. Phonophobia and brainstem excitability in migraine. Eur J Neurosci. (2021) 53:1988–97. 10.1111/ejn.15078 33305448

[24] Nayak S Parida M Das SB Padhi PK Behera M Patil A. Clinical characteristics and management of headache: a real-life prospective, observational study from a tertiary Care Center in Eastern India. Cureus. (2020) 12:e12409. 10.7759/cureus.12409 33409110 PMC7779133

[25] Sulena Singla M Brar J Kale R Kale S. Clinical profile of migraine in a rural population presenting to tertiary Care Hospital in North India. Ann Indian Acad Neurol. (2020) 23:781–6. 10.4103/aian.AIAN_671_19 33688127 PMC7900733

[26] Kalita J Uniyal R Bhoi SK. Is palinopsia in migraineurs an enhanced physiological phenomenon? Cephalalgia. (2016) 36:1248–56. 10.1177/0333102415625610 26767828

[27] Francis MV. Brief migraine episodes in children and adolescents-a modification to International Headache Society pediatric migraine (without aura) diagnostic criteria. Springerplus. (2013) 2:77. 10.1186/2193-1801-2-77 23526480 PMC3602609

[28] Menon B Kinnera N. Prevalence and characteristics of migraine in medical students and its impact on their daily activities. Ann Indian Acad Neurol. (2013) 16:221–5. 10.4103/0972-2327.112472 23956569 PMC3724079

[29] Mishra D Sharma A. Triggers of migraine in children at a public hospital in India. Springerplus. (2012) 1:45. 10.1186/2193-1801-1-45 23961370 PMC3725916

[30] Bhoi SK Kalita J Misra UK. Metabolic syndrome and insulin resistance in migraine. J Headache Pain. (2012) 13:321–6. 10.1007/s10194-012-0416-y 22278639 PMC3356472

[31] Gupta R Bhatia MS. Comparison of clinical characteristics of migraine and tension type headache. Indian J Psychiatry. (2011) 53:134–9. 10.4103/0019-5545.82538 21772645 PMC3136015

[32] Yadav RK Kalita J Misra UK. A study of triggers of migraine in India. Pain Med. (2010) 11:44–7. 10.1111/j.1526-4637.2009.00725.x 19793343

[33] Chakravarty A Mukherjee A Roy D. Trigger factors in childhood migraine: a clinic-based study from eastern India. J Headache Pain. (2009) 10:375–80. 10.1007/s10194-009-0147-x 19705059 PMC3452095

[34] Panda S Tripathi M. Clinical profile of migraineurs in a referral centre in India. JAPI. (2005) 53:111–5. 15847028

[35] Noor T Sajjad A Asma A. Frequency, character and predisposing factor of headache among students of medical college of Karachi. J Pak Med Assoc. (2016) 66:159–64. 26819160

[36] Bokhari FA Sami W Shakoori TA Ali SA Qureshi GA. Clinical characteristics of 226 college-going female migraineurs in Lahore, Pakistan - putting ICHD-2 to the road test. Neuro Endocrinol Lett. (2008) 29:965–70. 19112417

[37] Al-Shimmery EK. Precipitating and relieving factors of migraine headache in 200 Iraqi Kurdish patients. Oman Med J. (2010) 25:212–7. 10.5001/omj.2010.59 22043340 PMC3191636

[38] Al-Hashel JY Ahmed SF Alroughani R Goadsby PJ. Migraine among medical students in Kuwait University. J Headache Pain. (2014) 15:26. 10.1186/1129-2377-15-26 24886258 PMC4029817

[39] Hajj A Mourad D Ghossoub M Hallit S Geagea A Abboud H. Uncovering demographic, clinical, triggering factors similarities between migraine and irritable bowel syndrome: a prospective study. J Nerv Ment Dis. (2019) 207:847–53. 10.1097/NMD.0000000000001033 31503173

[40] Aljaafari D Aldossary N Almuaigel MF Alsulaiman FA Nazish S Zafar A. Migraine prevalence, characteristics, triggers, and coping strategies among medical students in Saudi Arabia. Prim Care Companion CNS Disord. (2021) 23:20m02859. 10.4088/PCC.20m02859 34592795

[41] Bamalan BA Khojah AB Alkhateeb LM Gasm IS Alahmari AA Alafari SA. Prevalence of migraine among the general population, and its effect on the quality of life in Jeddah, Saudi Arabia. Saudi Med J. (2021) 42:1103–8. 10.15537/smj.2021.42.10.20210575 34611005 PMC9129244

[42] Desouky DE Zaid HA Taha AA. Migraine, tension-type headache, and depression among Saudi female students in Taif University. J Egypt Public Health Assoc. (2019) 94:7. 10.1186/s42506-019-0008-7 30774147 PMC6351506

[43] Ibrahim NK Alotaibi AK Alhazmi AM Alshehri RZ Saimaldaher RN Murad MA. Prevalence, predictors and triggers of migraine headache among medical students and interns in King AbdulAziz University, Jeddah, Saudi Arabia. Pak J Med Sci. (2017) 33:270–5. 10.12669/pjms.332.12139 28523020 PMC5432687

[44] Özçelik P Koçoglu K Öztürk V Keskinoglu P Akdal G. Characteristic differences between vestibular migraine and migraine only patients. J Neurol. (2022) 269:336–41. 10.1007/s00415-021-10636-0 34109480

[45] Ur Özçelik E Lin K Mameniškienè R Sauter Dalbem J Siqueira HH Samaitiene R. Perceptions of modulatory factors in migraine and epilepsy: a multicenter study. Front Neurol. (2021) 12:672860. 10.3389/fneur.2021.672860 34149603 PMC8209378

[46] Akarsu EO Baykan B Ertaş M Zarifoglu M Kocasoy Orhan E Saip S. Sex differences of migraine: results of a nationwide home-based study in Turkey. Noro Psikiyatr Ars. (2020) 57:126–30. 10.293-99/npa.23240 32550778 PMC7285639

[47] Gur-Ozmen S Karahan-Ozcan R. Factors associated with insulin resistance in women with migraine: a cross-sectional study. Pain Med. (2019) 20:2043–50. 10.1093/pm/pnz055 30938814

[48] Mollaoglu M. Trigger factors in migraine patients. J Health Psychol. (2013) 18:984–94. 10.1177/1359105312446773 23104993

[49] Karli N Akgöz S Zarifoglu M Akiş N Erer S. Clinical characteristics of tension-type headache and migraine in adolescents: a student-based study. Headache. (2006) 46:399–412. 10.1111/j.1526-4610.2006.00372.x 16618256

[50] Aygül R Deniz O Koçak N Orhan A Ulvi H. The clinical properties of a migrainous population in eastern Turkey-Erzurum. South Med J. (2005) 98:23–7. 10.1097/01.SMJ.0000145390.12710.D5 15678636

[51] Karli N Zarifoglu M Calisir N Akgoz S. Comparison of pre-headache phases and trigger factors of migraine and episodic tension-type headache: do they share similar clinical pathophysiology? Cephalalgia. (2005) 25:444–51. 10.1111/j.1468-2982.2005.00880.x 15910569

[52] Köseoglu E Naçar M Talaslioglu A Cetinkaya F. Epidemiological and clinical characteristics of migraine and tension type headache in 1146 females in Kayseri, Turkey. Cephalalgia. (2003) 23:381–8. 10.1046/j.1468-2982.2003.00533.x 12780769

[53] Kececi H Dener S. Epidemiological and clinical characteristics of migraine in Sivas, Turkey. Headache. (2002) 42:275–80. 10.1046/j.1526-4610.2002.02080.x 12010384

[54] Bener A Uduman SA Qassimi EM Khalaily G Sztriha L Kilpelainen H. Genetic and environmental factors associated with migraine in schoolchildren. Headache. (2000) 40:152–7. 10.1046/j.1526-4610.2000.00021.x 10759915

[55] Okuma H Okuma Y Kitagawa Y. Examination of fluctuations in atmospheric pressure related to migraine. Springerplus. (2015) 4:790. 10.1186/s40064-015-1592-4 26702379 PMC4684554

[56] Katsuki M Tatsumoto M Kimoto K Iiyama T Tajima M., Investigating the effects of weather on headache occurrence using a smartphone application and artificial intelligence: a retrospective observational cross-sectional study. Headache. (2023) 10.1111/head.14482 36853848

[57] Al-Hashel JY Abokalawa F Toma R Algubari A Ahmed SF. Worsening of migraine headache with fasting Ramadan. Clin Neurol Neurosurg. (2021) 209:106899. 10.1016/j.clineuro.2021.106899 34464831

[58] Ragab AH Kishk NA Hassan A Yacoub O El Ghoneimy L Elmazny A. Changes in migraine characteristics over 30 days of Ramadan fasting: a prospective study. Headache. (2021) 61:1493–8. 10.1111/head.14231 34726767

[59] Schulte LH Peng KP. Current understanding of premonitory networks in migraine: a window to attack generation. Cephalalgia. (2019) 39:1720–7. 10.1177/0333102419883375 31615269

[60] Headache Classification Committee of the International Headache Society (IHS). The international classification of headache disorders, 3rd edition. Cephalalgia. (2018) 38:1–211. 10.1177/0333102417738202 29368949

[61] Schulte LH Jürgens TP May A. Photo-, osmo- and phonophobia in the premonitory phase of migraine: mistaking symptoms for triggers? J Headache Pain. (2015) 16:14. 10.1186/s10194-015-0495-7 25904144 PMC4385011

[62] Panconesi A. Alcohol and migraine: trigger factor, consumption, mechanisms. A review. J Headache Pain. (2008) 9:19–27. 10.1007/s10194-008-0006-1 18231712 PMC3476173

[63] World Health Organization. The Global Health Observatory; Alcohol, total per capita (15+*) consumption (in litres of pure alcohol) (SDG Indicator 3.5.2)*. Available online at: https://www.who.int/data/gho/data/indicators/indicator-details/GHO/total-(recorded-unrecorded)-alcohol-per-capita-(15-)-consumption?msclkid=fd317478b4ef11ec91943e4076cc3b77 (accessed February 18, 2022).

[64] Carod-Artal FJ Ezpeleta D Martín-Barriga ML Guerrero AL. Triggers, symptoms, and treatment in two populations of migraneurs in Brazil and Spain. A cross-cultural study. J Neurol Sci. (2011) 304:25–8. 10.1016/j.jns.2011.02.027 21402387

[65] Tai MS Yap JF Goh CB. Dietary trigger factors of migraine and tension-type headache in a South East Asian country. J Pain Res. (2018) 11:1255–61. 10.2147/JPR.S158151 29988763 PMC6029602

[66] Goadsby PJ Constantin L Ebel-Bitoun C Igracki Turudic I Hitier S Amand-Bourdon C. Multinational descriptive analysis of the real-world burden of headache using the Migraine Buddy application. Eur J Neurol. (2021) 28:4184–93. 10.1111/ene.15037 34309986 PMC9291858

