# Supplementary material for: Migraine triggers in Asian countries: a narrative review
Source: Front Neurol. 2023 May 3;14:1169795. doi: 10.3389/fneur.2023.1169795 (PMC10189151; doi:10.3389/fneur.2023.1169795)
Supplement: Supplementary Table 1 — Three most frequent triggers in each study. Age was not available in some studies. The provided information about the background of the studied group is shown below [age; mean (range)]. N/A, not available, (i) elementary/junior high school students, (ii) junior high school students, (iii) university students, (iv) medical students, (v) medical students and interns. [file Table_1.pdf]

Region	Year	First Author	Reference	Country	Age	N	Top 3 Triggers
Eastern	2018	Gu X	13	China	22.4 (18-35)	78	stress at study/work (94%), lack of sleep (92%), change in time of sleep (87%)
	2013	Wang J	14		41.7 (9-78)	394	sleep disturbance (40%),negative affect (34%), sunlight (33%)
	2017	Goto M	15	Japan	N/A i)	319	katakori (43%), fatigue (32%), lack of sleep (20%)
	2004	Takeshima T	16		N/A (20-)	244	fatigue (61%), lack of sleep (46%), mental stress (31%)
South-eastern	2020	Haw NJ	17	Philippines	31.6 (N/A)	511	stress/heavy workload (79%), looking at computer screens for too long (74%), lightning (50%)
	2010	Visudtibhan A	18	Thailand	13.2 (12-14)	116	inadequate sleep (22%), stress related to school activities (20%), excessive environment with bright light (9%)
	2010	Visudtibhan A	19		N/A ii)	28	academic stress (54%), lack of sleep (33%), computer related (13%)
Southern	2022	Rafi A	20	Bangladesh	21.9 iii)	503	stress (71%), irregular sleep (47%), academic pressure (33%)
	2016	Perveen I	21		21.1 iv)	51	stress (84%), lack of sleep (82%), frequent/prolong travelling (67%)
	2012	Haque B	22		N/A (N/A)	250	sunight (44%), stress (32%), anxiety (27%)
	2022	Kalita J	23	India	33.7 (N/A)	61	sun exposure (84%), noise (79%), odor (33%)
	2020	Nayak S	24		N/A (14-)	163	menstruation (46%), stress (39%), inadequate sleep (34%)
	2020	Sulena	25		36.8 (N/A)	1,245	stress (45%), noise (44%), excertion/sleep disturbance (36%)
	2016	Kalita J	26		32.2 (N/A)	65	mental stress (86%), sleep depriviation (79%), sun exposure (66%)
	2013	Francis MV	27		N/A (5-15)	1,402	exposure to sunlight (92%), travelling by bus (46%), strenous physical exercises like dancing and cycling (42%)
	2013	Menon B	28		N/A iv)	144	sleep disturbance (53%), weather/climate (49%), head movements (43%)
	2012	Mishra D	29		10 (3-17)	26	stress (42%), environmental noise (15%), lack of sleep (12%)
	2012	Bhoi SK	30		31.4 (14-65)	92	mental stress (96%), physical stress (95%), noise (93%)
	2011	Gupta R	31		27.7 (N/A)	50	noise (100%), light (80%), head bending (70%)
	2010	Yadav RK	32		30.7 (14-58)	182	emotional stress (62%), missed meal (41%), sleep deprivation (39%)
	2009	Chakravarty A	33		11.8 (7-15)	200	sunlight/hot and humid climate (94%), school stress (79%), environmental noise/crowded place (78%)
	2005	Panda S	34		30.4 (N/A)	198	travel (38%), tension (23%), hunger (22%)
	2016	Noor T	35	Pakistan	N/A iv)	86	anxiety (44%), hot weather (42%), loud noise (40%)
	2008	Bokhari FA	36		N/A (16-)	226	stress (35%), lack of sleep (18%), certain diet (2%)
Western	2010	Al-Shimmery E	37	Iraq	N/A (12-70)	200	sounds/psychological upset (80%), physical activity (68%), changes in weather (66%)
	2014	Al-Hashel JY	38	Kuwait	20.2 (16-25)	173	stress (25%), irregular sleep (21%), much reading (19%)
	2019	Hajj A	39	Lebanon	34.3 (10-66)	94	stress (81%), anxiety (77%), annoyance (72%)
	2021	Aljaafari D	40	Saudi Arabia	20.8 iv)	16	study reated stress (88%), emotional related stress (81%), sleep disturbance (75%)
	2021	Bamalan BA	41		29.6 (18-60)	766	sleep depriviation (94%), stress and anxiety (82%), sounds (79%)
	2019	Desouky DE	42		21.2 (22-26)	436	stress or anxiety (52%), irregular sleep (11%), menstruation (8%)
	2017	Ibrahim NK	43		21.5 v)	149	exam stress (83%), sleep disturbance (80%), emotional stress (73%)
	2021	Ozcelik P	44	Turkey	38 (23-70)	35	hunger (43%), noise (40%), emotional stress (26%)
	2021	Ur Ozcelik E	45		35.3 (15-83)	94	emotional stress (83%), negative feelings (61%), menstruation (58%)
	2020	Akarsu EO	46		36.9 (18-65)	871	stress (69%), wind (54%), hunger (54%)
	2019	Gur-Ozmen S	47		33.6 (18-65)	63	mental stress (76%), fasting<12h (71%), fasting＞12h/menstruation (70%)
	2013	Mollaoglu M	48		36.3 (18-65)	126	lack of sleep (64%), agressiveness (60%), hunger (54%)
	2006	Karli N	49		14.6 (12-17)	341	stress (76%), exercise (65%), sleeplessness (63%)
	2005	Aygül R	50		32.4 (8-64)	185	stress/tension (71%), changes in sleep (49%), noise (42%)
	2005	Karli N	51		38.1 (N/A)	56	stress-anxiety (80%), hunger (73%), menstruation (66%)
	2003	Köseoglu E	52		N/A (15-)	143	stress (82%), sleep deprivation (52%), fasting or starving (37%)
	2002	Kececi H	53		N/A (7-)	158	emotional stress (65%), fatigue (58%), sleeplessness (51%)
	2000	Bener A	54	UAE	N/A (6-14)	159	playing at the computer (46%), high noise (42%), hot climate (37%)
